# Effect of Chemical Blowing Agent on the PVC Cellular Coating Extrusion

**DOI:** 10.3390/ma13245752

**Published:** 2020-12-16

**Authors:** Tomasz Garbacz, Aneta Tor-Świątek, Tomasz Jachowicz

**Affiliations:** Faculty of Mechanical Engineering, Lublin University of Technology, 36 Nadbystrzycka Str., 20-816 Lublin, Poland; t.garbacz@pollub.pl (T.G.); t.jachowicz@pollub.pl (T.J.)

**Keywords:** poly(vinyl chloride), chemical blowing agent, coating extrusion, physical properties, mechanical strength

## Abstract

Depending on the type and application, the coatings of power, electric, telecommunication cables as well as other types of conduits are made of various kinds of polymer plastics. However, most often, because of good mechanical properties and many other advantages, they are first of all made from polyvinyl chlorine (PVC). This paper contains characteristics of the developed cellular extrusion of cable coatings, as well as specification of the blowing agent (BA) used and selected research results of the obtained cellular extrusion product. In technological tests the coating extrusion technological line was used. The material was modified with a new blowing agent of exothermic distribution of process characteristics, which was introduced into the material in quantities from 0.2 to 0.6% wt. The amount of blowing agent used has a direct impact on the density and structure of the received result for the extrusion of modified polymers. The cellular structure of the cellular coatings was presented. The results of the study are thin-walled properties of single- and double-layer cellular outer coatings, forming an outer surface on a steel wire. The research on the structure of manufactured materials, density and the degree of porosity, water and oil absorptivity, mechanical strength is presented.

## 1. Introduction

The technology of cellular extrusion of thermoplastics has in recent years been one of the faster-growing methods of processing of these materials. For the purpose of obtaining the right structure, those key properties of the extrudate need to be modified by using an apt polymer or introduction to the blowing agents (porophors) [1,2,3,4,5]. There are significant differences between the extrusion of cellular or solid plastics form. Therefore, this process gives the product of two-phase plastic-gas structured with possibly the smallest and most equally distributed gas bubbles [1,3,6,7,8]. The properties of a cellular extrudate not only depend on the type of plastic, porophor kind and content, dimensions, number and geometric characteristics of pores formed in the process, but also on the applied method and technology of the extrusion [1,9,10,11]. The structure is achieved by the insertion of the blowing agent (BA) in the form of an inert gas, low-boiling liquid or by inserting a solid body into the plastic input which, when in liquid or solid state, transforms into gas under the determined conditions of the extrusion process [9,12,13].

The blowing agents used in the extrusion process are characterized by an exothermic or endothermic decomposition process. BA’s main compositions are based on an organic acids, azodicarbonamide, hydrocarbons, sodium bicarbonate and nitrogen compounds. Therefore, they are called exothermic and endothermic BA [9,12,13,14,15]. The use of chemical blowing agents in the processing process requires setting the appropriate temperature in the plasticizing system so that the decomposed temperature of the BA is exceeded at a specific point in the plasticizing system. Whilst the time of the material flow through the system and the extrusion die is as long as possible. Therefore, the gas can be released in the right amount [1,8,9,12,14,16]. The gases generated in the plasticizing system are mostly air, nitrogen and carbon dioxide. These gases mix and dissolve in the plastic, creating numerous cells (pores) of various shapes and sizes [9,10,16,17,18,19]. The obtained cells keep enlarging until gas pressure and interfacial tension are balanced [20,21,22].

The condition for obtaining the favourable cellular structure of the polymer in the extrusion process is the proper temperature distribution through the plasticizing system of the extruder and the extrusion dies. Temperature in the first, second and optionally the third functional zone of the plasticizing system should have a value lower than the temperature of the beginning of the decomposition of the BA. In the last section of the plasticizing system, the temperature should exceed the decomposition temperature of the agent, and together with the dies temperature should be close to the decomposition temperature of the blowing material or lower [1,11,17,18,21].

The extrusion speed also has a significant influence on the qualities of the cellular result. At low extrusion speed, the foaming process takes place in the nozzle of the extrusion head and after the polymer leaves the extrusion head, which results in thermal instability of the process and requires the use of special calibration systems and further intensive cooling [18,19,22,23]. Depending on the cooling temperature, a different porosity distribution of the structure of the extrudate can be achieved; the cellular products take longer to set than those of solid products. Although the heat capacity of the cellular material is lower, its heat transfer greatly deteriorates. The cooling of the cellular extrudate (the range and intensity) in a decisive way influences the creation of the cellular structure of the extrudate [18,23,24,25].

The extruded material can be entirely solid or cellular, cellular in the whole mass, or it can have a cellular core as well as a solid surface. The properties of cellular extrudate depend on both, the type of a polymer, and on the blowing agent type and content, the size, number and geometry of cells produced in extrusion, as well as effectiveness of the cellular extrusion process [9,15,26,27,28].

This cellular process causes a product of different properties to be received and takes effect in the decrease in the cost of purchase of polymers, the increase in the extrusion efficiency, the lower cost of energy of the process as well as lower transport costs in the course of manufacturing [16,21,29,30]. As an end-product, introducing the blowing agent with the polymer during the cellular processing, the obtained cellular product has new, modified physical, technological and functional properties. These are, e.g., lower density, little processing shrinkage, better damping and insulating properties, lower flammability and water and oil absorptivity [9,17,29,30,31,32,33,34,35].

The main purpose of the work presented in the article was to get to know the influence of polyvinyl chlorine (PVC) modification with the selected blowing agent on the course and effectiveness of the production of porous products in the extrusion process. A further goal of the work was to obtain cellular coatings with satisfactory physical and functional properties, appropriate geometric properties, as well as meeting market requirements. To best understand the nature of the changes taking place in the porous material, the density and porosity of the extruded products were tested. The aim of the research was also to find out about the mechanical resistance and adsorption resistance of the coatings, which are representative functional properties determining the use of cellular single- and double-layer coatings on steel wires, including for the production of shielding nets. An analysis of the porous structure was also carried out, the aim of which was to determine the state of the porous layer, its size, and pore distribution, which also influences the identification of the correlation from the physical and mechanical characteristics of the cellular coatings.

The research on the technology of producing thin-walled steel wire coatings during the cellular extrusion of PVC modified with a blowing agent is completely original. A novelty is the analysis of the effectiveness of the production of cellular coatings and their use in the production of coated wire mesh fencing. There are also no tests of the physical, functional and structural properties of thin-walled PVC coatings of steel wire. This makes it impossible to compare with the results of research obtained by other authors related to the issues of processing cellular plastics and its properties.

New application possibilities and the need to improve the quality of coated products, such as wire mesh fencing, cables and wires of various types and applications, cause constant interest in the possibilities of technology modification and properties of coated products among enterprises and factories. The obtained research results will allow us to gain new knowledge in the field of processing and qualities of thin-walled steel wire and cable coatings. The advantage resulting from the production of thin-walled coatings with the use of BAs is the new, modified physical and functional abilities of the products and the new possibilities of their use [4,7,9,19,21,28]. By using a blowing agent into the material in the extrusion also provides significant material and energy savings of this process, reducing production costs.

## 2. Materials and Methods

The items used in the experiment included polyvinyl chloride (PVC), one type of chemical blowing material. PVC manufactured by Anwil SA (Przemyśl, Poland), known by its trade name EPC-1, previously examined on cellular extrusion process. Those PVCs with density ranging from 1230 to 1400 kg/m^3^ and hardness of 80 °Sh A, MFR_(150 °C/1029 kg)_ vary from 4.3 and 4.6 g/10 min, with a tensile strength of ≥21 MPa (acc. to the information provided by the producer). The recommended processing temperature for this material is between 120 and 190 °C.

PVC was modified with an original chemical blowing agent, which was introduced into the material in an amount of 0.2 to 0.6% depending on its weight, i.e., 0.2, 0.4 and 0.6%, respectively. It is an agent constituting a material system under the trade name Hydrocerol 530, produced by Clariant Masterbatch Division, in the form of granulate, wherein the carrier of the blowing agent is PVC. Hydrocerol 530 as a blowing material has exothermic characteristics of the decomposition process with nucleating abilities. It comes in a granular form, with grains ranging between 2.4 and 2.8 mm. In order to achieve higher foaming process efficiency, the processing degree should range between 150 and 170 °C. Active substances in this BA contain a mixture in the right proportion of chemical compounds for instant azodicarbonamide. Accordingly with research program, the introduction of BA into PVC was achieved through the mechanical mixing process.

**Figure 1:**
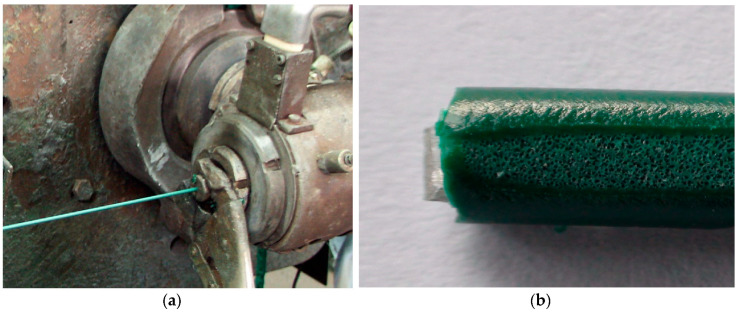
Fragment of the produced metallic wire with a polyvinyl chlorine (PVC) coating: (**a**) Producing coated wire with a single layer coating in an amount of 0.4% of a blowing agent (BA); (**b**) Fragment of the longitudinal section of the double coatings PVC + 0.6% BA.

The process for producing the outer coating of wire, with the use of a polymer modified with a blowing agent, was carried out using the extrusion coating wire technology line. A production line was used to produce single-layer outer coating and a line used to produce double-layer outer coating. The technological line for the extrusion of the wire coating consisted of a main single-screw extruder (D = 45 mm, L/D = 25) and an auxiliary single-screw extruder (D = 25 mm, L/D = 25), an extrusion cross-die to the coatings (Figure 1), a device for cooling, transporting and winding the extrudate.

The processing temperature ranges were, respectively: 150, 160, 170 and 185 °C in the plasticizing system of the extruder and 160, 150 °C in the extrusion die. The temperatures were used so that the decomposition of the BA occurs in the other half of the plasticizing system and extrusion die. The obtained extrudate was cooled in a cooling device at a temperature of 16 to 20 °C. It is vital that the process of the decomposition of the porophor under the influence of the temperature/heat, would start at the last phase of the plasticizing system and endure in an uninterrupted way for the extrusion die.

As a result of the conducted cellular extrusion process PVC containing blowing agent, the product obtained is in the form of a coated wire with the outer layer and double layer with a solid outer surface and cellular core.

In line with recommendations and in accordance with proper standards, density, porosity, water and oil absorptivity in relation to the extruder product obtained from cellular PVC were examined.

To determine the density of the samples of the PVC extrudate, the immersion method was applied [36]. The measurements were conducted on the fragmented particles of coatings samples, the mass of which was in the range from 1 to 5 g. The determination of water and oil absorbance was carried out according to the requirements of the relevant standard ISO 62:2008 and ISO 175:2002 [37,38]. A core in the form of a metallic wire is taken out of the cut samples, and then appropriate samples of single- and double-layer coatings were placed in distilled water and mineral oil with a temperature of 23 °C ± 2 °C, first for 48 h ± 1 h and then for 168 h ± 1 h.

The study and analysis of the cellular structure of the extrudates were carried out, by using an ultra-high accuracy digital microscope, type Keyence VHX 7000, SEM microscopy (Tescan Vega/LMU from Brno, Czech Republic) and stand for testing the structure of polymer materials. The Microscope VHX 7000 was equipped with 4 K CMOS image sensor, allowing direct preview of the observed structure on the screen.

To determine selected mechanical properties of the obtained thin-layer wire coatings, a study was performed for tensile strength together with elongation at the break. The applied shape and size of test samples was in accordance with the relevant standard [39]. For the measurement, the coating samples were used after removing the steel wire. Measurements were carried out using a Zwick/Roel Z010 from Germany testing machine. Tensile speed 10 mm/min, measuring load in ranges of 0 ÷ 500 N and the accuracy of measuring the strength of ±1 N.

## 3. Results

**Figure 2:**
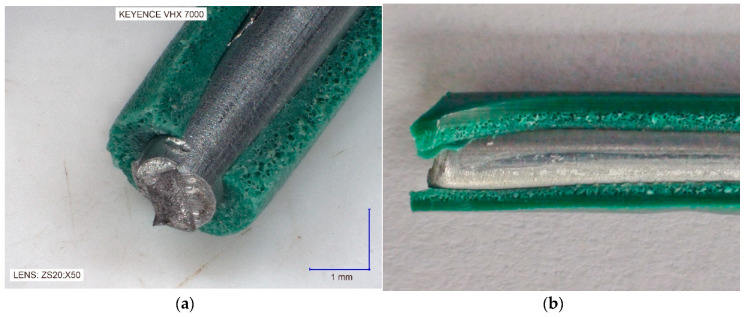
Fragment of the longitudinal section of the metallic wire with PVC cellular coating: (**a**) Single coating; (**b**) Double coating.

As a consequence of the coating PVC extrusion containing BA, the product obtained is in the form of a coated wire with two types of thin-walled coating. It was a coated wire with a single layer coating a solid surface layer, an inner cellular layer (Figure 2a).

The obtained coated wire had a product diameter of 2.60 mm with a coating thickness of 0.60–0.70 mm, in accordance with relevant industry standards.

In the case of a coated wire with a double-layer coating, with the inner cellular coating and the outer solid coating, the coated wire had the outer diameter of 3.80 mm and total coating thickness of 0.70–0.80 mm (Figure 2b).

**Figure 3:**
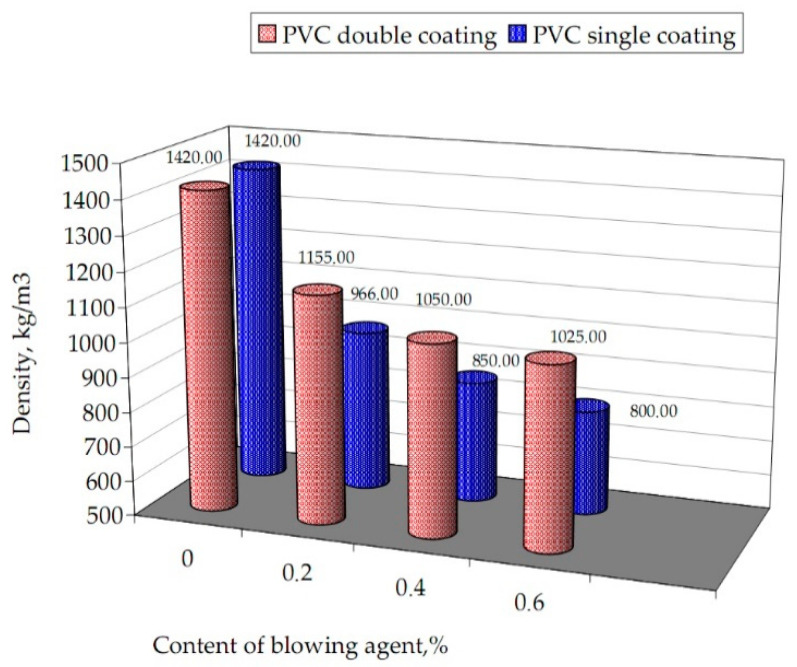
Dependence of density of the coatings with PVC on the content of BA.

In the conducted studies on cellular extrusion processes, the type and quantity of the proportioned blowing agent was selected in such a way that under determined processing conditions, cellular coatings with a solid topcoat and cellular core were obtained, with apparent density varying from 1155 to 800 kg/m^3^ (Figure 3). The obtained wire coating degree of porosity ranged from 19 to 40%, respectively (Table 1). This value defines the percentage of cellular surface in relation to the total surface of the tested coating.

The single layered coatings made using a PVC with an exothermic blowing agent showed a density in the range of 966 kg/m^3^ (by 0.2% BA) to 800 kg/m^3^ (by 0.6% BA). The density of double layered coatings specimens changed from 1155 to 1025 kg/m^3^ at the blowing material amount from 0.2 to 0.6%, respectively. Cellular coating from PVC were characterized by porosity in the range of 32 to 40% and from 19 to 28% with BA content ranges 0.2–0.6%, respectively.

Table 1 summarized liquid absorption. The single layered coatings made using a PVC with exothermic BA showed a water immersion in the range of 1.6% (0.2% blowing agent content) to 2.15% (0.6% blowing agent content) given 48 h and from 2.35 to 3.10%, after 7 days.

The absorption of double layered coatings specimens changed from 1.50 to 2.80% (after 48 h) and from 2.15 to 3.10% (after 168 h) at the BA in a range of 0.2% to 0.6%, respectively. Cellular coatings from PVC were characterized by oil absorption in the range of 0.35 to 0.80% (after 48 h) and from 0.60 to 1.50% (after 168 h) with the blowing agent content in the range of 0.2 to 0.6%, respectively.

The oil absorption of cellular coatings increases from 0.35 to 1.50% with the BA amount from 0 to 0.6%. The double layered coatings on metallic wire characterized water absorptivity from 1.5 (0.4% wt. BA, 48 h.) to 2.8% (0.6% wt. BA, 48 h.) and from 2.65 to 3.60%, after 168 h. The double layered coatings were characterized by analogous changes in water absorptivity.

The consequences of chosen mechanical properties of single layer and double layer coatings, obtained with different content of blowing agent, are represented in Table 2.

**Figure 4:**
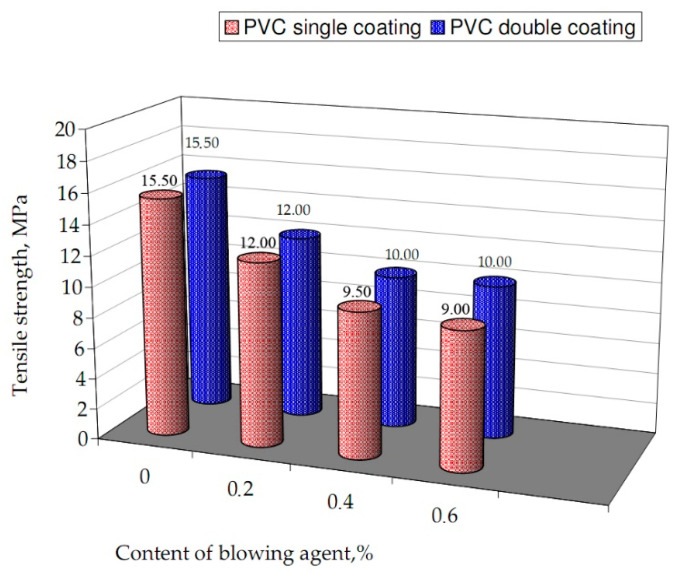
Dependence of tested cellular coatings tensile strength on the content of BA.

The obtained results of tensile tests are presented as a graphical form. Figure 4 shows examples of the relation between stress at break and the contents of blowing agent in the material. It was observed that with an increase in the degree of porosity of the product, the value of break stress decreased with a growing intensity. Elongation increases non-linearly, together with the degree of porosity of the coatings, throughout entire range. For instance, Hydrocerol 530 0–0.6% decreased tensile strength by 40% in single layer coatings (Table 2). In the case of double layer coatings with up to 0.4% content of BA, the tensile strength decreases on average by 35% in extruded coatings. For 0.6%, the tensile strength equals 10.0 MPa, which decreases for unmodified PVC by 35% (10 MPa).

**Figure 5:**
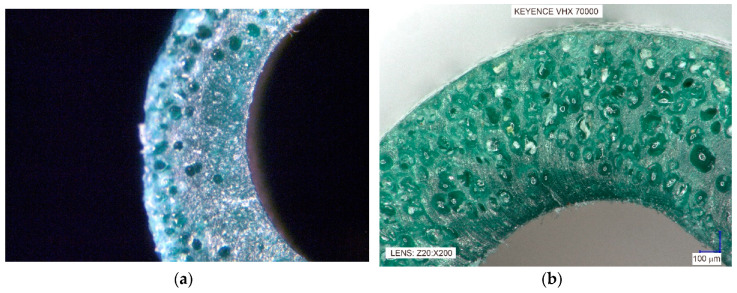
Part with the cross section for the single coatings: (**a**) PVC + 0.2% BA; (**b**) PVC + 0.6% BA.

**Figure 6:**
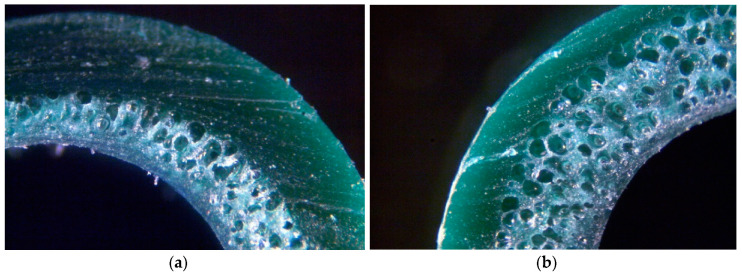
Fragment of the cross section of of the double coatings: (**a**) PVC + 0.2% BA; (**b**) PVC + 0.6% BA.

**Figure 7:**
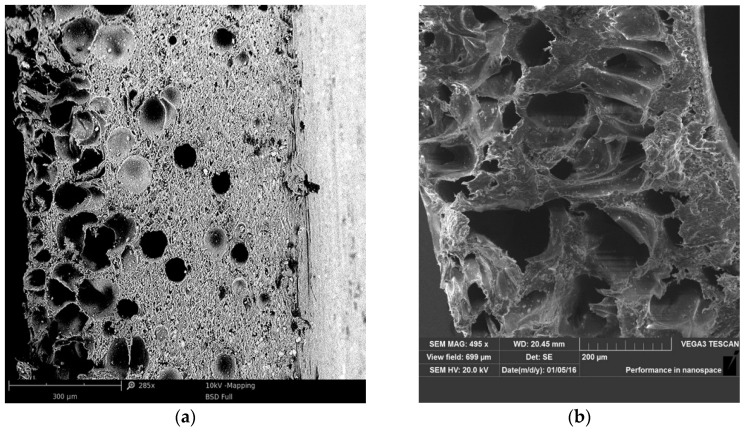
Scanning electron micrographs of the PVC single coatings: (**a**) PVC + 0.2% BA; (**b**) PVC + 0.6% BA.

**Figure 8:**
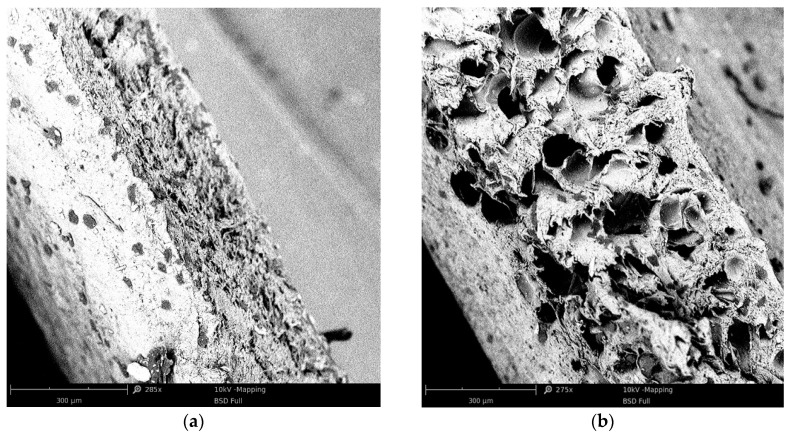
Scanning electron micrographs micrographs of the PVC double coatings: (**a**) PVC + 0.2% BA; (**b**) PVC + 0.6% BA.

The produced coatings are shown in Figure 5, Figure 6, Figure 7 and Figure 8. The views of the cross section were taken on the digital microscope, SEM microscope and an optical tooling for a polymer cellular structure image equipped with a digital camera and a computer with suitable software.

The BA was added in 0.2–0.6% in order to produce appropriate coating for a metallic wire with a solid surface and a cellular core. The shape and outside dimensions of the product correspond with the shape and dimensions of solid coatings made of the homogenous PVC.

PVC coatings made with 0.2% exothermic blowing agent amount does not have sufficient cellular structure (Figure 5a and Figure 7a). The photos show that single layer coating samples (Figure 5 and Figure 7) are porous in the entire area. Pores differ in size and shape. The single layered coating of 0.6% content of the blowing agent (Figure 5b and Figure 7b), is characterized by a cellular structure of a different size and shape of the obtained pores. This may result in the formation of a too thin solid outer layer, and as a result, possible ruptures and discontinuities.

There is a substantial concentration of pores of various sizes which, in extreme cases, can cause a discontinuity in the solid outer layer. There is no solid solid outer layer visible, as in the case of double layer coatings.

The porosity of a double layered coating made of PVC + 0.2% wt. BA is far too small. The cellular structure is characterized by a tiny number of pores of various shapes and sizes. This is due to the small amount of BA in the material and a possible inaccurate dosing or mixing of so little BA (Figure 7a).

The porosity of the double layered coating containing 0.6% wt. BA is adequate, and the distribution of the pores in the cross-section is more uniform. The resulting cellular structure is characterized by a large number of pores of a more uniform shape and size (Figure 6b and Figure 8b).

The pores of the largest size are in the center of the cellular layer, while the pores of the smaller size are in the layer near the surface, directly next to the solid layer of the produced coating (Figure 7 and Figure 8). This is due to the speed and cooling efficiency of the cellular coating. Effective cooling prevents and reduces the growth of pores located directly at the surface of the coating.

## 4. Discussion

The properties of cellular coating, taking into consideration criteria like porosity and density, should be assessed as very good. The obtained product has an outer coating with a degree of porosity from 19 to 40%, and density from 1155 to 800 kg/m^3^, depending on the amount of the BA in the modified materials.

The density of porous coatings decreases as the amount of BA in the PVC increases. This is consistent with the state of the art [1,9,11,19], which found that the density also largely depends on the quantity of the solid and gas phases with the shape and size of the pores. The decomposition of azodicarbonamide, which is a blowing agent, at an appropriate temperature causes the formation of numerous microbubbles, which immediately dissolve in the surrounding material due to pressure and surface development [1,10,16,19,35]. The resulting pores are filled mostly with air, but also other gases, causing PVC to expand in volume. As a result, the obtained coatings are reduced in density. Regardless of the type of coatings, the porosity of the coatings increased depending on the increase in the pore-forming agent dosed, which is the result of a change in coating density [5,22,34].

In the conducted research, the greatest effective change of porosity occurs whilst adding BA to PVC, in the amount of 0.4% wt. The obtained porosity for a single layered and a double layered coating is 24 and 38%, respectively. The content of 0.2% wt. BA in PVC creates a structure with porosity 19%, which is limited (/too little) porosity, resulting in too little savings in the processed PVC.

Cellular materials made using a PVC with exothermic BA showed a water absorption varying from 1.6% (by 0.2% blowing agent content) to 2.15% (by 0.6% blowing agent content). For 48 h and given 7 day ranges from 2.35 to 3.10%. Water absorption changes for dual layer coatings analogically. The oil absorption of coatings specimens changed from 0.25 to 1.70% at the blowing agent content ranging from 0% to 0.6%, respectively. In some practical applications this may be too large to accept.

However, in most of the tested cases, in the consequences of liquid absorption tests are consistent with the operational requirements of the tested coated wire mesh fencing. According to the test results and their analysis [8,17,22,34], both the water absorption and the weight gain after immersion in oil do not change rapidly with the increase in the blowing agent content in the processed material. This may prove that the pores formed in the porous extrusion process are closed and prevent the ingress of water and oil into the porous extrudate.

With increased amount of BA in the PVC the tensile strength decreases, together with yield strength and elongation. The elongation at the break decreases monotonically with the amount of BA in the material tested. Those dependencies are analogous for each of the single layered and double layered coating. It is related to the characteristics of the resulting cellular structure. It has mostly closed pores which initiates the material cracking during strength tests.

Represented relationships are partially justified in the analyzed literature [2,7,21,28], confirming that the mechanical properties of the modified material are influenced by the direction shape, and bonds of macromolecules. As it results from the cited literature [4,14,27,32], materials with linear macromolecules and chains similar to each other due to crystallization or orientation show greater strength than porous materials in the interaction of inner forces at the boundary of the solid phase and gas phase is reduced. The amount of this agent introduced directly affects the density and structure of the obtained extrudate, while these parameters further affect the tested mechanical properties.

Based on the analysis of the cellular structure, it was determined by a single layer of coatings with 0.2–0.4% wt. amount of BA (Figure 5a and Figure 7a) have a distinct, solid outer layer, which differ in shape and size, and their distribution is irregular.

For a double layered coating with 0.2% blowing agent, the porosity value is too low. The plate structure of the porous varies in shape and size. This may be due to uneven mixing of the amount of blowing agent in the PVC (Figure 7a). Effective surface cooling stops the growth of pores. Therefore, the largest number of pores with the smallest size exists closer to the solid body layer.

This has a fragmentary reference to the state of literature [3,7,13,21], in which the change in pore size occurring in individual areas could be affected by the intensity of cooling of the product. The fastest cooling of the porous product took place in its outer layer, which prevented further expansion of the BA. Extrudating the core, the cooling intensity was lower, allowing for further pore growth, which was presented in the studies on the production of the outer coating of electric cables [5,22,34]. The use of blowing materials with exothermic properties produces a product with an irregular porous structure. In the literature [15,20,29,33] it was found that this is related to the nature of the action of this type of material. Initiated exothermic degradation of the blowing agent proceeds in an unsustainable way. The process will last even after the heat is completely cut-off. Therefore, the cell products have a heterogeneous cell structure.

## 5. Conclusions

The cellular extrusion process, using chemical blowing agents, allows us to make coatings with new, modified properties without much interference in the process technology. Changing these properties offers new possibilities for using these types of products while reducing the cost of producing coatings.

The quality of the product, and analyzed properties, for instance a porosity and a density, should be assessed as very good. The obtained single-layer and double-layer coatings of steel wire do not have visual changes related to the properties of the geometric structure of the coating.

Based on research results, it has been found that the preferred content of the blowing agent in PVC should be 0.6% wt. in such an amount of agent results in a structure with a porosity of approx. 40% and an even distribution of pores of a similar shape and size.

The results of liquids absorption showed even greater absorptivity of the porous materials, depending on the time spent in the tested environment. Cellular PVC coatings meet the requirements of the appropriate recommendations regarding the properties of PVC coatings on metallic wires. According to the standards, satisfactory water and oil absorptivity is 3–4% for various types of coatings.

Those mechanical properties of PVC materials, including their mechanical impact strength, depend on the cellular structure of the material and pore orientation. It was found that the characteristics of the poring agent distribution affect the properties of the coating materials studied. The agent added to 0.4–0.6% contributes to a significant decline in the mechanical properties of the examined materials based on PVC.

It was assumed that for a satisfactory reduction in the mass of the PVC product, whilst lowering the mechanical strength within the acceptable range, the amount of the BA in the processed PVC cannot exceed 0.4%. This is related to the cost-effectiveness of the cellular process and the recommendations regarding the used properties of coatings on metallic wires.

The advantage of products made in the process of cellular extrusion, which are coatings of wires and cables, is their porous structure, which results in savings in polymer material, by 30% or even up to 40%, needed for their production.

## Figures and Tables

**Table 1:** Results of porosity, liquid absorption of PVC coatings.

Coating Type	Porosity %	BA Content, %	Water Absorption, %		Oil Absorption, %	
			48 h	168 h	48 h	168 h
Solid	0	0	2.20	2.60	0.55	0.70
Single layer	32	0.2	1.60	2.35	0.55	0.90
	38	0.4	1.80	2.85	0.60	1.30
	40	0.6	2.15	3.10	0.80	1.50
Double layer	19	0.2	1.50	2.15	0.35	0.60
	24	0.4	2.30	2.50	0.50	1.10
	28	0.6	2.80	3.10	0.80	1.40

**Table 2:** Research results of the mechanical properties of coatings, average values with accuracy 0.5 MPa.

Coating Type	BA Content, %	Tensile Strength MPa	Yield Strength MPa	Elongation at Break %
Solid	0	15.5	14.0	not break
Single layer	0.2	12.0	11.0	236
	0.4	9.5	9.0	220
	0.6	9.0	8.5	210
Double layer	0.2	12.0	10.5	256
	0.4	10.0	9.0	250
	0.6	10.0	9.0	250
